# Exploring meaning in life from social network content in the sleep scenario

**DOI:** 10.3389/fpubh.2025.1642085

**Published:** 2025-11-11

**Authors:** Qi Li, Mengyao Wang, Junjie Yan, Wu Jiake, Liang Zhao, Xin Wang, Bowen Yao, Lei Cao

**Affiliations:** 1 Beijing Key Laboratory of Applied Experimental Psychology, National Demonstration Center for Experimental Psychology Education (Beijing Normal University), Faculty of Psychology, Beijing Normal University, Beijing, China; 2 School of Information Management, Wuhan University, Wuhan, China; 3 Institute of Biomedical Engineering, University of Oxford, Oxford, United Kingdom; 4 School of Economics and Management, Beijing Jiaotong University, Beijing, China

**Keywords:** meaning in life, social network, factor extraction, semantic analysis, deep learning

## Abstract

**Introduction:**

The exploration of life’s meaning has been a key topic across disciplines, and artificial intelligence is now beginning to investigate it.

**Methods:**

This study leveraged social media to assess meaning in life (MIL) and its associated factors at individual and group levels. We compiled a diverse dataset consisting of microblog posts (*N* = 7,588,597) and responses from user surveys (*N* = 448), annotated using a combination of self-assessment, expert opinions, and ChatGPT-generated insights. Our methodology examined MIL in three ways: (1) developing deep learning models to assess MIL components, (2) applying semantic dependency graph algorithms to identify MIL associated factors, and (3) constructing eight subnetworks to analyze factors, their interrelations, and MIL differences.

**Results:**

We validated these methods and bridged two foundational MIL theories, highlighting their interconnections.

**Discussion:**

By identifying psychological risk factors, our work may provide clues to mental health issues and inform possible intervention.

## 1 Introduction

Meaning in life (MIL) has long been recognized as the sense that one’s life has purpose, coherence, and significance (1). Building on this view, later research typically distinguishes the Presence of Meaning in Life (POM) and the Search for Meaning in Life (SFM) (2). From a Self-Determination Theory perspective, MIL has been linked to autonomy, competence, and relatedness needs (3). Higher MIL was related to greater life satisfaction (4, 5) and better health indicators (2). It was found to protect against psychological distress (6), depression (7–9), anxiety (67), and loneliness (10). It also related to mortality-related concerns, including death anxiety and suicide risk (10–12). Beyond mental health, MIL was linked to better sleep quality (13), lower risk of eating disorders (14), and enhanced coping among individuals with chronic conditions such as cancer and HIV (15, 16).

Building on the importance of MIL, our research question concerns how MIL can be assessed. Existing assessments have relied on validated questionnaires developed from diverse theoretical perspectives. These included the Purpose in Life questionnaire (17), the Meaning in Life Questionnaire with Presence and Search subscales (2), and the Meaningful Life Measure (18). Subsequently, the Multidimensional Existential Meaning Scale conceptualized comprehension, purpose, and mattering (19). Most recently, the Three-Dimensional Model of Meaning conceptualized coherence, purpose, and significance (20). Although these tools have advanced our understanding of MIL’s structure and correlates, two research gaps remain. First, because MIL is abstract and multifaceted, there is no universally accepted structured definition, and the relations between competing frameworks such as two-dimensional and three-dimensional models require further clarification. Second, while questionnaires capture subjective perceptions well, they are limited in scalability, timeliness, and adaptability for large-scale assessment in naturalistic settings.

This study introduces a novel approach to automatically assess an individual’s MIL from social media expressions and examines differences in linguistic patterns across POM and SFM levels. Three lines of evidence support the feasibility of this approach. First, MIL could emerge from everyday experiences such as establishing daily routines (21, 22), and online interactions often mirrored real-world social dynamics (23, 24). Second, social media content provided a window into existential reflection (25, 26). Moreover, social media engagement was closely associated with processes of meaning making (23). More active engagement such as posting photos was related to a stronger sense of purpose in life (25, 27), whereas passive browsing without interaction mediated the relation between meaning and self-esteem in stressful contexts (28). Third, on the technical side, prior studies inferred implicit psychological variables from user-generated content (29), including anxiety (30), insomnia (31, 32), stress (33–35) and stressors (36), and suicide risk (37).

Building on these foundations, we moved from usage patterns to language-based, large-scale assessment of MIL in social media text. We then examined how levels of POM and SFM relate to associated factors at the group level. This study aims to address two central research questions:

*RQ1*: The relationship between POM and SFM is complex and sometimes inconsistent. How can we model these two components from real-world social media data?

*RQ2*: The associated factors that shape MIL are varied and often interrelated (e.g., achievements, security, spirituality, health, family life). How can we accurately extract these factors from large-scale textual data and characterize their interconnections?

Specifically, this study assessed MIL within the sleep context for three reasons. First, on theoretical grounds, prior work showed that rumination and self-evaluation occurred more often at night (38). Moreover, rumination was closely associated with MIL (39). Ge (31) further showed that POM significantly predicted sleep quality via mediators such as depression. These studies supported sleep as a theoretically dense window on MIL. Second, from a methodological perspective, constraining the analysis of textual expressions of MIL to the sleep context reduced noninformative noise in open social media data. It increased the density and retrievability of MIL-relevant signals, which made the linguistic features easier to capture and model. Third, from a reproducibility perspective, using sleep as a contextual starting point facilitated replication across platforms. The methods could be reapplied with matched time windows in follow-up studies.

**Figure 1:**
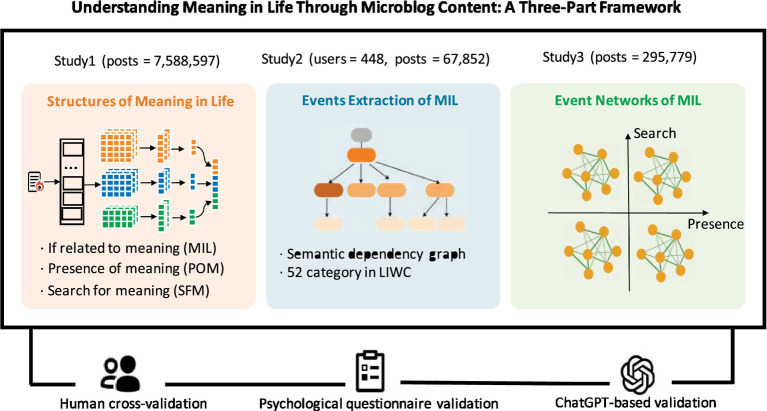
Three-part framework for exploring MIL from microblog content.

Focusing on the sleep context, we collected over 7,500,000 microblogs, of which 189,213 contained MIL-related keywords. We conducted manual annotation and data augmentation on sampled posts, constructing a labeled dataset comprising 3,000 MIL-relevance labels, 1,600 POM (High vs. Low) labels, and 1,600 SFM (High vs. Low) labels. The three-part framework is shown in Figure 1. In Study 1, we developed three binary deep learning models to assess MIL relatedness, POM level, and SFM level. We then applied these models to segment the large-scale microblog dataset into eight subgroups reflecting different MIL states. Study 2 employed semantic dependency graphs to extract associated factors and their semantic relations. In Study 3, we constructed eight subnetworks to support downstream analyses of associated-factor patterns, including their effects on MIL, their interconnections, and differences across subnetworks.

This study makes contributions both methodologically and theoretically. Methodologically, we introduce a framework for automated, timely assessment of MIL and its associated factors. Beyond scalable analysis of social media content, the framework has practical implications. It supports early detection of mental health risks. For example, it can monitor loss of meaning as a precursor to depression or insomnia. It also helps tailor interventions in educational or occupational settings and informs the design of digital platforms that enhance social support and well-being. Theoretically, we classify the associated factors into five areas: factor frequency, influential factors, factor relationships, significant factor differences, and clustering trends, and explore their implications for the two-component model of MIL, offering new insights into the complexities of meaning.

## 2 Methods

### 2.1 Dataset

**Figure 2:**
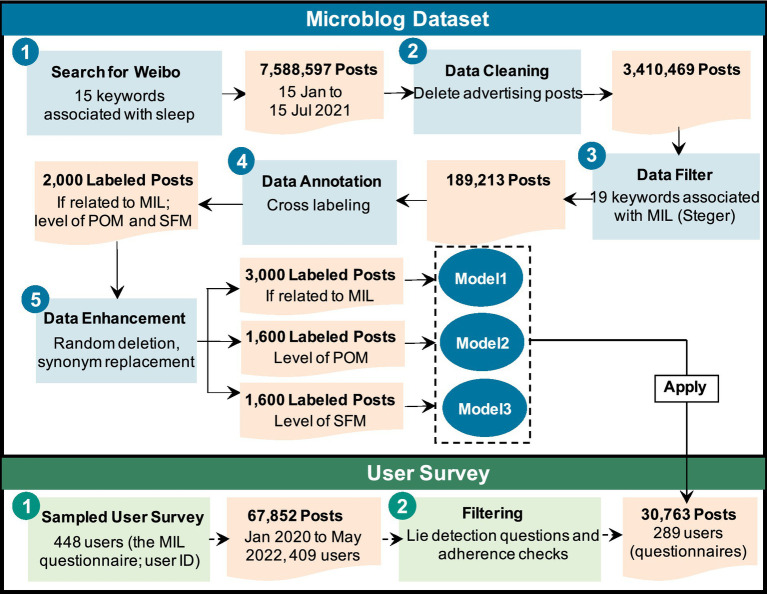
Dataset construction process: microblog dataset and user survey.

To address the absence of publicly available datasets for MIL, we started by constructing a reliable corpus comprising two parts: a large-scale Weibo dataset and an empirical dataset collected through participant recruitment (see Figure 2).

#### 2.1.1 Microblogs

We executed a Python program on Sina Weibo, a leading social media platform in China, between December 2021 and January 2022. It collected 7,588,597 sleep-related microblogs posted from January 15 to July 15, 2021 (see Figure 2). All collected posts were originally written in Chinese. The sleep-related seed keywords/expressions were chosen based on two criteria: (1) terms related to sleep derived from the Pittsburgh Sleep Quality Index (40) and (2) synonyms of these terms identified in the microblog corpus. The seed list of sleep-related keywords/expressions included insomnia, staying up late, having many dreams, nightmare, waking up startled, sleepy, early morning, having a dream, unable to fall asleep, sleep, easy to wake up, and dreaming about. The criteria for selecting seed keywords/expressions related to MIL were as follows: (1) identifying terms associated with MIL from the Meaning in Life Questionnaire (16); and (2) selecting synonyms of these keywords/expressions found in the microblog text. The MIL seed list included meaning, purpose, value, faith, ideal, aspiration, future, pursuit, quest, seek, establish, presence, and exploration.

In this study, posts were retained in their original Chinese form throughout the entire processing pipeline, including preprocessing (data cleaning and augmentation), word embeddings, and modeling, without intermediate translation that might introduce semantic distortion. An initial review revealed substantial noise, such as advertisements and *http* links. We employed a stop-word list containing 469 entries to filter out noisy posts. Examples were provided in Supplementary Table A1. The complete list is available at the “Dataset and Code” link (see Data Availability). This list comprised common Chinese function words, punctuation marks, and high-frequency non-informative terms identified in the corpus [e.g., terms related to advertising and fan-engagement super-topic hashtags (#)]. In addition, we removed duplicate texts longer than 10 Chinese characters. After cleaning, the dataset comprised 3,410,469 posts.

This dataset was then screened using the MIL seed keywords/expressions, yielding 189,213 posts that constituted the high-density MIL dataset. From this subset, we randomly selected 2,000 posts, divided them into 10 files, and conducted cross-annotation with the assistance of 10 psychology undergraduates. For each post, the annotator performed three binary classification tasks: (1) determine if the post is “Related” or “Not Related” to MIL or “Unable to Judge”; (2) for MIL-related posts, assess as “High POM” or “Low POM” or “Unable to Judge”; and (3) for MIL-related posts, assess as “High SFM” or “Low SFM” or “Unable to Judge.” Detailed examples and annotation guidelines are provided in Supplementary Tables B1, B2. Specifically, for MIL we excluded 532 posts in total (184 with inconsistent labels and 348 labeled as Unable to Judge), leaving 1,468 consistently labeled posts (68.8% Related, 31.2% Not Related). Within the 1,010 MIL-related posts, we excluded 176 with inconsistent SFM labels and 154 labeled as Unable to Judge, leaving 680 posts for SFM (63.4% High, 36.6% Low). For POM, we excluded 207 with inconsistent labels and 181 labeled as Unable to Judge, leaving 622 posts for POM (49.4% High, 50.6% Low). Cohen’s kappa was 0.84 for MIL, 0.71 for SFM, and 0.68 for POM (all *p* < 0.01).

Data augmentation was then applied to the consistently labeled dataset (excluding Unable to Judge cases) using two strategies: random deletion and random synonym replacement. Random deletion involved removing one or two noncritical words (for example, adverbs or intensifiers) without altering the central meaning. Random synonym replacement substituted words with semantically close synonyms (cosine similarity >0.80 using Sentence-BERT embeddings). To ensure semantic validity, a random 10% of the augmented posts was manually checked by two annotators, and items with altered meaning were discarded. After augmentation, the dataset was expanded to 3,000 posts for Model 1 (MIL), and 1,600 posts each for Model 2 (SFM) and Model 3 (POM).

#### 2.1.2 Participants

We recruited Sina Weibo users through two channels: the AiShiyan Participant Recruitment Platform and the Weibo Super Topic “#Questionnaire#.” This resulted in 222 users and 701 users signing up, respectively, for a total of 923 participants. Informed consent was obtained from all participants. They were asked to complete the Meaning in Life Questionnaire (2) and to provide their user ID. Among them, 448 individuals completed the questionnaire. We then defined active users as those who posted more than 10 original microblogs between January 1, 2020, and May 4, 2022. We filtered out these active users and also removed prominent marketing accounts, which were characterized by exceptionally high numbers of comments, likes, and shares per post. After these exclusions, the dataset included 409 users and 67,852 posts. We excluded participants who failed the lie-detection item. The instruction was: “Please select ‘Strongly Agree’ for this question.” We also excluded patterned responses (i.e., selecting the same option for all items). This yielded 315 valid participants and 55,476 microblogs.

Finally, we applied the cleaning rules described in section 2.1.1. Specifically, we filtered posts containing advertisements, *http* links, or items from the customized stop-word list, and we removed duplicate posts longer than 10 Chinese characters. After cleaning, we retained 289 active users and 30,743 posts, averaging 106.38 posts per user and 44.25 characters per post. Detailed demographics were listed in Supplementary Table E3. The overall process is illustrated in Figure 2.

### 2.2 Study 1: a three-stage process for structurally assessing MIL from social media text

This study implemented a three-stage pipeline to assess MIL from microblog text. Model 1 detected whether a post was related to MIL. Model 2 classified posts into high or low levels of SFM. Model 3 classified posts into high or low levels of POM. In each stage, we adopted Bidirectional Encoder Representations from Transformers (BERT) (41) as the primary encoder to convert tokenized text into contextual representations. After encoding, a Text Convolutional Neural Network (TextCNN) (42) applied multiple convolution filters to capture the significance of keywords and enhance the models’ ability to identify key semantic cues. The input for all three models was raw post text, and the output was class probabilities and a predicted label.

**Figure 3:**
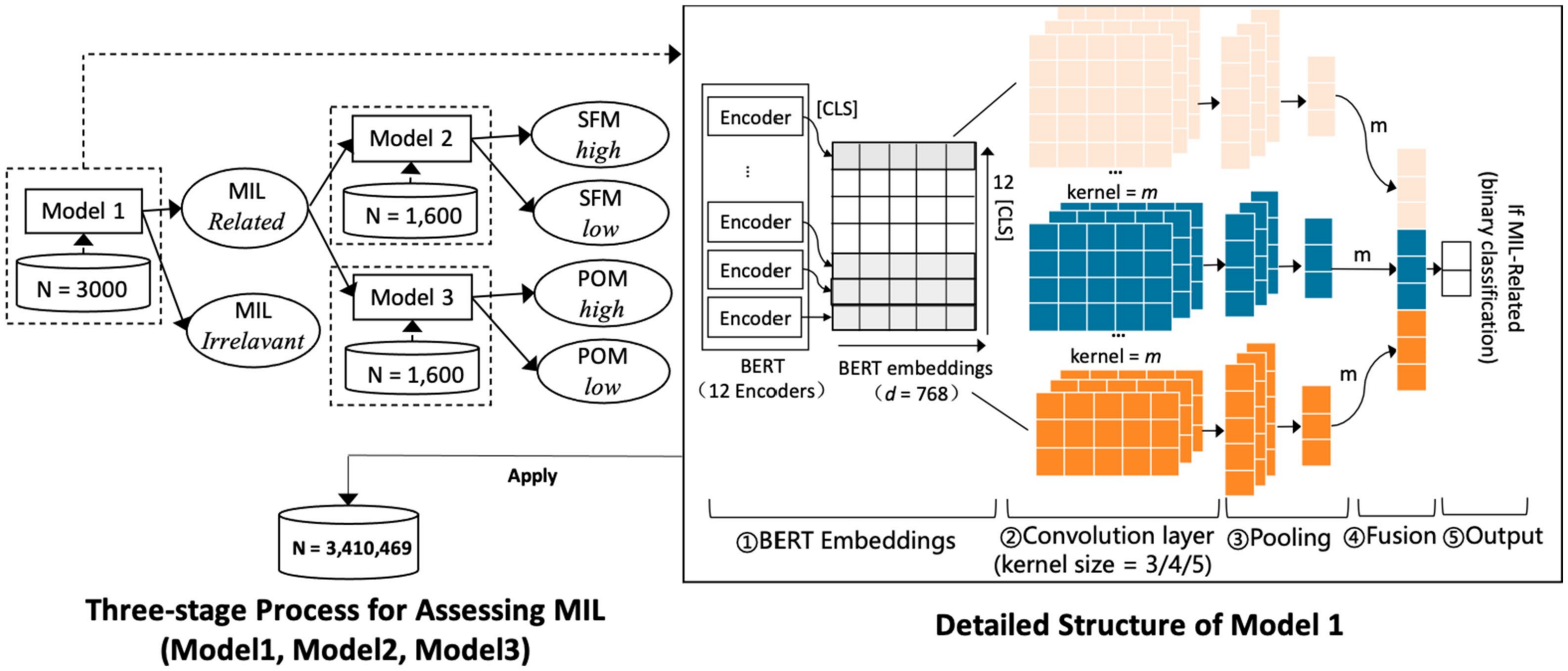
Structures of the three MIL assessment models. Model 1 determines whether a post is MIL-related (Yes/No). If a post is MIL-related, Model 2 evaluates the SFM level (High/Low) and Model 3 evaluates the POM level (High/Low). Detailed symbol definitions are provided in section 2.2.

The three models shared a five-layer architecture, as illustrated in Figure 3. The first layer was the BERT encoder with 12 transformer blocks. Shallow blocks captured lower-level semantics and deeper blocks encode higher-level semantics. For each input, the classification token vector (CLS; i.e., the representation of the special [CLS] token used by BERT) from each block was extracted and stacked to form a matrix that served as the input to the convolutional module. The second layer was the convolutional layer. Three sets of convolution filters with window sizes of *3 × d*, *4 × d*, and *5 × d* were applied, where *d* denoted the dimensionality of the BERT embeddings (typically 768) and *m* was the number of filters for each window size. These filters slid over the stacked [CLS] matrix to extract local features and generate one-dimensional feature maps. The third layer was the pooling layer, which applied max pooling to reduce dimensionality and retain the most informative signals. The fourth layer was the fusion layer, which concatenated the pooled outputs from the three window sizes into a single vector. The fifth layer was the output layer, where a fully connected layer followed by the softmax function predicted task-specific class probabilities.

The annotated microblog dataset was randomly divided into training, validation, and test sets in an 8:1:1 ratio. Each microblog was tokenized and mapped to subword units with special tokens added, and sequences were truncated or padded to a length of 164. Training used a batch size of 32 and a learning rate of $2\times 10^{-5}$ , and was performed using a 10-fold cross-validation approach to ensure robust model evaluation and prevent overfitting. During training, prediction errors were calculated, and the parameters were iteratively adjusted using the Adam optimizer (43). Early stopping was applied when validation performance did not improve for 1,000 update steps. Class imbalance was handled with class weights in the loss function. The validation set was used to tune model hyperparameters, and the test set was used for the final evaluation of model effectiveness. Using identical architecture and training settings across the three tasks ensured comparability of results.

### 2.3 Study 2: identifying associated factors of MIL through semantic dependency graph algorithms

We used the Language Technology Platform (LTP) Python package (44) to construct semantic dependency graphs. These graphs were intended to collect elements associated with MIL and to illustrate potential co-occurrence and directional tendencies among them, not causal pathways. We began from such associations to identify semantic cues that explained why something happened, which was relevant to MIL. We then mapped the extracted elements to Linguistic Inquiry and Word Count (LIWC) categories to enable standardized comparisons across groups.

As input to the LTP package, each post was segmented into linguistic units (i.e., sentences ending with a period, exclamation mark, or question mark), and the output was a set of dependency relations labeled with semantic roles. LTP provided a neural pipeline for Chinese that performed sentence segmentation, part-of-speech tagging, dependency parsing with a graph-based parser, and semantic role labeling. We focused on nine key semantic dependency roles: reason (REAS, i.e., the cause or motivation behind an action), agent (AGT, i.e., the entity performing the action), experiencer (EXP, i.e., the entity that perceives or experiences an event), object (PAT, i.e., the entity that is affected by the action), content (CONT, i.e., the subject matter or information conveyed), dative (DATV, i.e., the recipient of something in a transaction), link (LINK, i.e., a relationship or connection between entities), temporal (TIME, i.e., the time at which an action occurs), and locative (LOC, i.e., the place where an action occurs). Each dependency role was represented as a three-tuple <(wordA, wordB), Role>.

**Figure 4:**
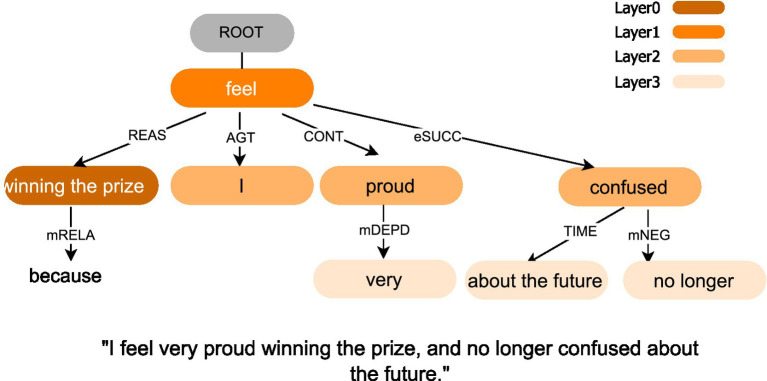
Examples of semantic dependency graphs of microblog texts. The color transition from dark to light indicated the relationship between the current role and REAS role, moving from closer to more distant connections. The nine semantic roles are reason (REAS), agent (AGT), experiencer (EXP), object (PAT), content (CONT), dative (DATV), link (LINK), temporal (TIME), and locative (LOC). ROOT represents the virtual root node that anchors the sentence’s semantic head (main predicate).

As shown in Figure 4, the outputs of LTP were iteratively expanded from the REAS role (Layer 0) to subsequent layers (Layers 1–3), thereby constructing the semantic dependency graph and identifying relationships between semantic roles. Nodes in the graph were lexical items normalized to surface forms after tokenization. Edges were added when two nodes were linked by any of the nine roles within a sentence. Edge weights were the corpus counts of such links aggregated across sentences and posts, and self-links were removed. For readability and robustness, we filtered stop words and punctuation and pruned edges with very low frequency. This graph served as the foundation for detecting associated factors and mapping them to LIWC categories. A high-level pseudocode of this process was provided in Supplementary Table C1.

Next, we reviewed the LIWC Chinese Dictionary, and extracted keywords (associated components) from the semantic dependency graphs that were indicative of MIL, thereby mapping the associated factors to their corresponding LIWC categories. The step-by-step pseudocode and the definitions of variables and symbols were provided in Supplementary Tables C2, C3. We adopted 52 LIWC categories and consolidated them into nine broader groups (see Supplementary Table D1).

### 2.4 Study 3: multi-level network analysis of associated factors triggering MIL

To examine the associated factors identified in Study 2 at a higher level of community detection, we conducted network analysis based on 295,777 posts containing MIL-related associated factors. All posts were divided into eight sub-datasets based on POM (High/Low) and SFM (High/Low) levels, corresponding to eight subnetworks. Four sub-networks captured single-dimensional variations (High SFM, Low SFM, High POM, Low POM), while the other four reflected the joint distribution of both dimensions (High POM and High SFM, High POM and Low SFM, Low POM and High SFM, Low POM and Low SFM). This design allowed us to analyze how different levels of SFM and POM, individually and jointly, shaped the manifestation of MIL in social media.

In each subnetwork, nodes represented associated factors extracted from semantic dependency graphs in Study 2, while edges represented correlations between these factors. Edge strength was measured using Pearson correlations, and the extended Bayesian information criterion was applied to identify connections and prevent overfitting (45). Centrality index, particularly expected influence, was used to quantify the relative importance of each node by accounting for both the magnitude and direction of connections (46). The bootnet package in R (Version 1.5.0) (46) was used to estimate the network structure, and the qgraph package (Version 1.9.2) (47) was used to visualize the networks and calculate centrality.

Furthermore, we applied the Louvain method (48) to cluster factors within each subnetwork across different SFM and POM levels. In the Louvain method, a *community* referred to a group of nodes (factors) that were more densely connected within the group than with nodes outside. *Density* was defined as the ratio of the number of observed edges (*E*) to the maximum possible number of edges among *N* nodes, i.e.,

$$\text{Density}=\frac{2E}{N(N-1)}$$

*Modularity* quantitatively measured the quality of a partition by comparing the density of within-community connections to the density expected under a random graph that preserved node degrees. Its standard definition is:

$$Q=\frac{1}{2m}\sum_{i,j}(A_{\mathrm{ij}}-\frac{k_{i}k_{j}}{2m})\delta (c_{i},c_{j})$$

where $A_{\mathrm{ij}}$ is the adjacency matrix, $k_{i}$ and $k_{j}$ are the degrees of nodes i and j , m is the total number of edges, and $\delta (c_{i},c_{j})$ is 1 if nodes i and j are in the same community and 0 otherwise.

The procedure comprised three steps: (1) measured link density within communities, (2) clustered nodes to maximize modularity, and (3) merged communities and repeated the process until modularity no longer improved, thereby optimizing the partition of the MIL network.

## 3 Results

### 3.1 Performance and applications of MIL assessment models

#### 3.1.1 Performance of MIL assessment models

We integrated BERT with TextCNN and fine-tuned three binary classifiers for the three-stage MIL assessment. Model 1 detected whether a post was related to MIL. Model 2 classified posts into high or low levels of SFM, and Model 3 classified posts into high or low levels of POM. Results (Table 1) indicated that the three models performed well, with consistently high validation and test accuracies (all above 88%). Model 1 achieved a validation accuracy of 93.67% and a test accuracy of 93.67%, demonstrating robust performance in the binary classification of MIL-related content. Model 2 reached a validation accuracy of 88.75% and a test accuracy of 90.62%, reflecting its effectiveness in capturing individuals’ varying levels of SFM. Model 3 also achieved strong results, with a validation accuracy of 95.00% and a test accuracy of 93.12%, confirming its reliability in distinguishing between high and low levels of POM. Taken together, these findings support the feasibility of our modeling approach for assessing MIL and its two core components from social media texts. This performance is conditional on the constructed dataset. We will then evaluate the approach on larger-scale datasets.

**Table 1:** Performance of three MIL assessment models in microblogs.

Model 1 [evaluating if the post is related to MIL (yes/no)]									
Training and testing (*N* = 3,000)						Applying in large scale microblog datasets (*N* = 3,134,657)			
Train loss	Train acc.	Val loss	Val acc.	Test loss	Test acc.	Posts (related)	Posts (unrelated)	Acc.(human-labeled)	Acc.(ChatGPT)
0.05	96.88%	0.24	89.33%	0.2	93.67%	295,777(9.44%)	2,838,880(90.56%)	90.40%	64.10%

Model 2 [judging the level of SFM (high/low)]									
Training and testing (*N* = 1,600)						Applying in large scale microblog datasets (*N* = 3,134,657)			
Train loss	Train acc.	Val loss	Val acc.	Test loss	Test acc.	Posts (high SFM)	Posts (low SFM)	Acc.(human-labeled)	Acc.(ChatGPT)
0.21	96.88%	0.33	88.75%	0.24	90.62%	158,804(53.69%)	136,972(46.31%)	74.90%	41.00%

Model 3 [judging the level of POM (high/low)]									
Training and testing (*N* = 1,600)						Applying in large scale microblog datasets (*N* = 3,134,657)			
Train loss	Train acc.	Val Loss	Val acc.	Test loss	Test acc.	Posts (high SFM)	Posts (low POM)	Acc.(human-labeled)	Acc.(ChatGPT)
0.11	96.88%	0.19	95.00%	0.18	93.12%	138,453(46.81%)	157,324(53.19%)	75.00%	47.50%

We further compared the model performance with two commonly used deep learning models for natural language classification tasks: (1) Long Short-Term Memory (LSTM) and (2) Enhanced Representation through Knowledge Integration (ERNIE). Taking Model 1 as an example, as shown in Table 2, the “BERT + TextCNN” model achieved a precision of 0.92, recall of 0.89, and an F1 score of 0.90, outperforming other models on the same dataset. For instance, standalone BERT had a lower F1 score of 0.85, and “BERT + LSTM” and ERNIE achieved F1 scores of 0.60 and 0.72, respectively.

**Table 2:** Performance of the MIL assessment model (model 1) compared with baseline deep learning methods.

Model	Precision	Recall	F1-score
BERT	**0.95**	0.76	0.85
BERT+LSTM	0.70	0.52	0.60
ERNIE	0.77	0.68	0.72
BERT + TextCNN	0.92	**0.89**	**0.90**

Bold values indicate the highest performance among models.

#### 3.1.2 Applications to a large-scale microblog dataset

We then applied the MIL assessment models to a large corpus of 3,410,469 microblog posts. We constructed a stratified random sampling frame to validate the models using human cross-validation and ChatGPT-based validation (Table 1). First, Model 1 identified 295,777 MIL-related posts (8.67%) from the full corpus; Models 2 and 3 then assessed MIL levels (High vs. Low) for SFM and POM. Posts were categorized into four groups: High SFM and High POM, High SFM and Low POM, Low SFM and High POM, and Low SFM and Low POM. These groups were used for associated factor extraction and MIL network analysis. Second, for these 295,777 MIL-related posts, we built semantic dependency graphs (section 2.3, Study 2) and obtained 43,338 posts (14.65%) with identified associated factors and LIWC categories (Table 3). Third, from this 43,338-post frame, we drew a stratified sample of approximately 1% (*n* = 462) with at least 100 items per SFM × POM quadrant. This size follows common practice for large-corpus quality checks, yielding an overall 95% CI with a half-width of approximately 5 percentage points and, per quadrant, approximately 9 to 10 percentage points, balancing coverage and annotation cost. The realized composition was High SFM and High POM: 102 (0.88%), High SFM and Low POM: 111 (1.22%), Low SFM and High POM: 134 (2.22%), and Low SFM and Low POM: 115 (0.69%). In the validation sample, the marginal distributions were SFM (High 46.1%, Low 53.9%) and POM (High 51.1%, Low 48.9%), indicating how the two components were represented.

**Table 3:** Associated factor extraction for MIL: algorithm performance.

Dimensions	Category (top 1)			Category (top 3)			Examples of factor extraction	
	Acc. (model)	Cohen’s kappa	Acc. (GPT)	Acc. (model)	Cohen’s kappa	Acc. (GPT)	Semantic dependency	Categories (top 3)
Low SFM and low POM (*N* = 16,620)	0.6	0.337	0.581	0.85	0.494	0.640	Now, during sleepless nights often caused by anxiety, a sense of inner loss frequently leaves me feeling a lack of belonging. (anxiety → sleepless, REAS), (belonging → feeling, CONT), (now → leaves, TIME), (loss → leaves, EXP), (nights → leaves, TIME), (me → feeling, AGT)	Biology, present tense, negative
Low SFM and high POM (*N* = 6,043)	0.703	0.314	0.489	0.766	0.536	0.596	My unremarkable life was also the happiest, marking the most beautiful memories. (memories → making, LINK), (my → happiest, EXP), (unremarkable → happiest, REAS)	Compare, consciousness, positive
High SFM and low POM (*N* = 9,133)	0.586	0.589	0.564	0.859	0.662	0.663	I have my own life plans and aspi_x0002_rations, but often lose motivation because of family entanglements. (family → entanglements, EXP), (motivation → loss, LINK), (entanglements → loss, REAS), (plans → have, LINK)	Drive, achieve, social
High SFM and high POM (*N* = 11,542)	0.351	0.408	0.441	0.846	0.571	0.577	I feel very proud winning the prize, and no longer confused about the future. (feel → winning the prize, REAS), (feel → I, AGT), (feel → proud, CONT), (confused → about the future, TIME)	Achieve, positive, compare
Total	0.56	0.412	0.519	0.83	0.566	0.619	–	–

“Top 1” denotes the highest-ranked factor per post; “top 3” denotes the set of the three highest-ranked factors per post. “Category” refers to the LIWC category mapped from keywords identified in the semantic dependency graph.

Building on the sampling procedure described above, we then used the random subset of 462 posts from the 43,338 posts with identified associated factors to conduct a manual validation by four psychology students and a ChatGPT-based validation. Specifically, we implemented a Python program that accessed the *gpt-3.5-turbo* model via the OpenAI API. The detailed prompt was provided in Supplementary Table E1. This prompt used a one-shot prompting strategy. An example post was presented first to instruct the model on the task. Each new post in the out-of-sample subset was then evaluated with the same prompt. Both the manual labels and the assessment results of our models (Models 1, 2, and 3) were blinded to ChatGPT. We reported *joint-label accuracy* in Table 1, which requires the SFM and POM labels to be correct simultaneously. In manual validation, Model 1 achieved 90.40% accuracy, Model 2 achieved 74.90%, and Model 3 achieved 75.00%. In the ChatGPT-based validation, accuracies were 64.10, 41.00, and 47.50% for Models 1, 2, and 3, respectively.

In addition, the user-survey validation showed a significant correlation between MIL scores and the proportion of MIL-related posts (*p* = 0.006), further supporting the effectiveness of Model 1 (see Supplementary Table E2). This analysis was conducted among participants who had posted MIL-related content, as identified by Model 1.

### 3.2 Results of semantic dependency graphs and associated factors in MIL

#### 3.2.1 Performance of the associated-factor extraction algorithm

For the 295,777 MIL-related posts identified by Model 1, we built semantic dependency graphs and extracted 43,338 instances with identified associated factors and corresponding LIWC categories. Each post could contain multiple associated factors across LIWC categories. LIWC categories were ranked by frequency (descending). The highest-ranked was labeled Top 1, and the top three were labeled Top 3. Posts were categorized into four groups based on SFM and POM, with examples shown in Table 3. Manual cross-validation was conducted on the sampled dataset described in section 3.1.2 (*n* = 462) by four psychology students. Their evaluation assessed whether the Top 1 category matched the MIL associated factor (Cohen’s kappa = 0.412) and whether the Top 3 categories covered the factor (Cohen’s kappa = 0.566). With manual cross-validation, our algorithms achieved Top-1 and Top-3 accuracies of 0.560 and 0.830. With ChatGPT-prompt validation, the corresponding accuracies were 0.519 and 0.619 (Table 3).

#### 3.2.2 Proportions of associated factors across eight MIL groups

**Figure 5:**
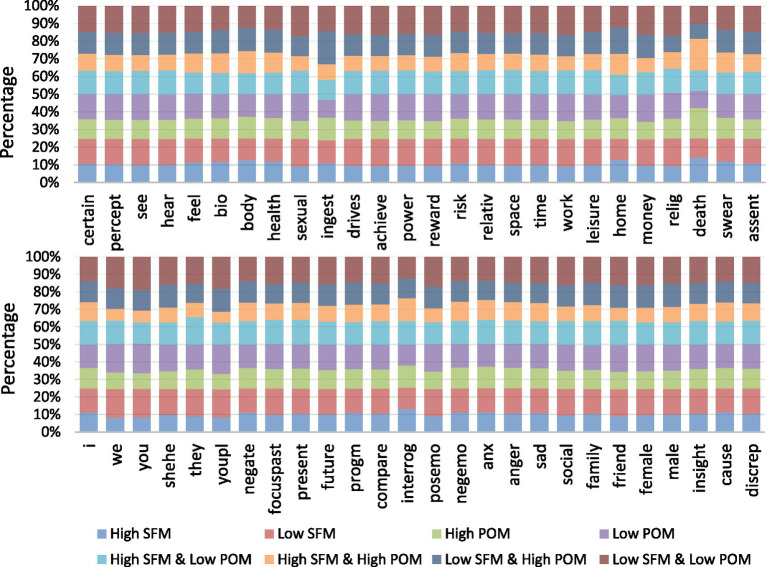
Proportions of associated factors within eight MIL groups by LIWC category (*n* = 52).

We examined the distribution of 52 LIWC Chinese Dictionary categories across the eight groups defined by levels of POM and SFM (Figure 5). Several patterns emerged. First, groups with higher POM and lower SFM showed higher proportions of everyday activity categories. For example, terms related to daily routines (e.g., “ingest” and “eat”). Second, groups with lower POM and lower SFM showed higher proportions of second-person address, reflected in the frequent use of “you.” Third, the Interrogation category was most frequent in SFM High posts, including terms such as “when” and “what.” Finally, death-related content appeared more often in groups with higher POM. Among the eight groups, High SFM and High POM and High POM showed the highest rates for the LIWC Death category at 15.08 and 14.33%, respectively.

### 3.3 Multi-level network analysis of associated factors in MIL

After dividing MIL-related posts into eight sub-datasets by POM (High/Low) and SFM (High/Low) levels, we first presented a centrality analysis to identify influential factors (section 3.3.1). We then showed pairwise relationships highlighting the strongest co-occurrences between factors (section 3.3.2). Finally, we compared structural patterns across subnetworks (section 3.3.3), focusing on clustering differences and network-wide contrasts.

#### 3.3.1 Centrality analysis of nodes influencing MIL

**Figure 6:**
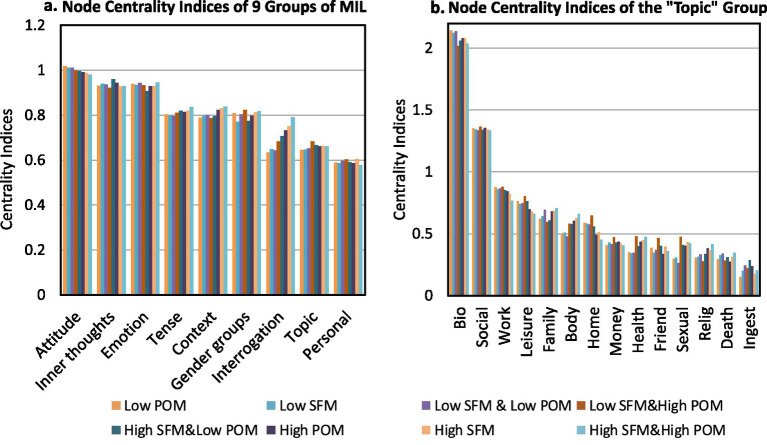
Node centrality indices of eight MIL subnetworks. **(a)** Node centrality indices of 9 groups. **(b)** Node centrality indices of the “Topic” group. For example, in panel **(a)**, the “Attitude” category shows the highest node centrality values across all subnetworks, suggesting that it contains many factors that play key bridging roles in MIL expressions (e.g., “assent” and “compare”). Additional factors in this category are listed in Supplementary Table D1.

**Figure 7:**
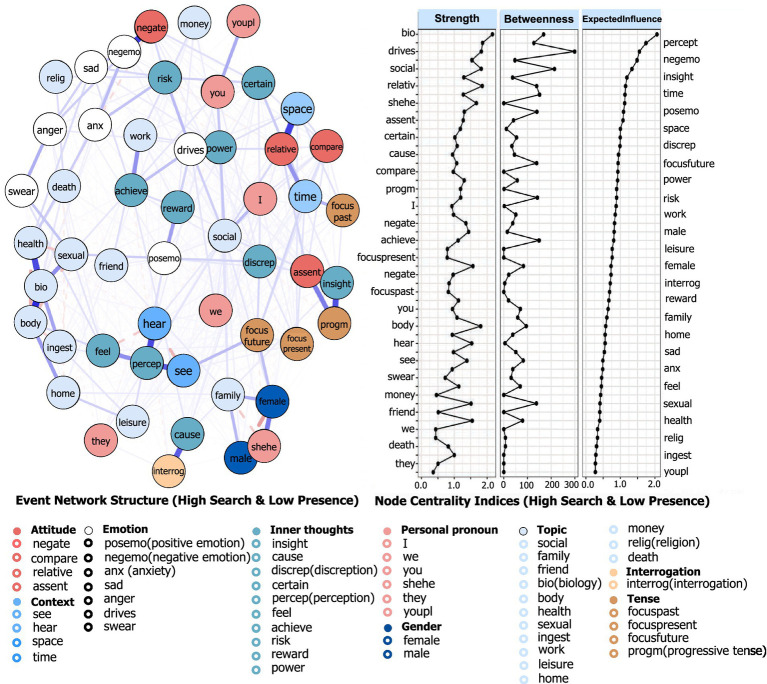
MIL-factor network structure and node centrality: using the high SFM & low POM subnetwork as an example. Each node corresponds to one of the 52 LIWC categories, aggregated into nine groups, each shown in a different color. Thicker edges indicate greater edge strength. For example, the nodes with the highest centrality are “bio,” “drive,” and “perception,” suggesting that these factors play a stronger connective role in shaping individuals’ MIL expressions in this state.

This section aimed to identify which factors function as key nodes within each subnetwork and to highlight differences in node centrality across subnetworks. The 52 associated factors were further grouped into nine higher-level categories to reveal more concentrated and interpretable regularities (see Figure 6). Node centrality indices in the High SFM and Low POM subnetwork were shown as an example in Figure 7. The “Attitude” category exhibiting the highest node centrality indices (indicating the greatest influence), followed by “Emotion” and “Inner Thoughts.” This suggested that these categories contained numerous factors that served as key bridges in MIL expressions (e.g., “assent” and “compare”). Detailed factors were listed in Supplementary Table D1. Within the “Topic” category, “Bio” emerged as the most influential, underscoring the critical role of physical state in shaping MIL.

The complete results for the 52 factors across the eight subnetworks were presented in Supplementary Table F1. Key findings included. (1) Across all subnetworks, the nodes with higher centrality were “bio,” “perception,” “drive,” “negative emotion,” and “social.” (2) “Shehe” ranked higher in the High SFM and Low POM subnetwork (node centrality index = 1.13). (3) “Social” and “work” showed their highest centrality in Low SFM and High POM (node centrality index = 1.37). (4) “Positive emotion” and “religion” rank highest in High SFM and High POM (node centrality indices = 1.20 and 0.41, respectively). (5) “Achieve” was highest in High SFM contexts (node centrality index = 0.82). (6) “Compare” showed higher centrality in Low POM subnetworks than in the other subnetworks (node centrality index = 0.96).

#### 3.3.2 Latent relationships among associated factors in MIL

We highlight the strongest pairwise co-occurrences among MIL-associated factors and illustrate how these relationships differ across subnetworks. The top 15 correlations for each subnetwork were listed in Supplementary Tables F2, F3, with the strongest pairs shown in boldface. The full 52 × 52 matrices were available at the “Dataset and Code” link (see Data Availability). Highlighted structures for all subnetworks were shown in Supplementary Figures F1, F2. Each node represented a LIWC category. Thicker edges and shorter inter-node distances indicated stronger correlations between categories. Key observations included. (1) Across all conditions, “space” and “time” were strongly correlated with the “relative” theme in MIL discussions. (2) In the High POM panel of Supplementary Figure F2, the “body” and “bio” nodes were close and connected by a thick edge. Consistent values were observed, with *r* = 0.779 for Low SFM and High POM and *r* = 0.810 for High SFM and High POM (both *p* < 0.001) (Supplementary Tables F2, F3). (3) In the Low POM panel of Supplementary Figure F2, the “power” and “drive” nodes were close with a thick edge. Corresponding values were observed, with *r* = 0.783 for Low SFM and Low POM and *r* = 0.790 for High SFM and Low POM (both *p* < 0.001) (Supplementary Tables F2, F3).

#### 3.3.3 Structural comparison of MIL subnetworks

We contrasted network-wide structures across the eight subnetworks, emphasizing clustering differences as well as global strength and weight invariance. The structural comparisons were presented in Supplementary Figure F3. The results indicated the following. (1) Across MIL levels, “space” and “time” appeared in different clusters: in SFM High and POM Low, “space” and “time” clustered with “focus past,” “focus present,” and “focus future”; in SFM Low and POM Low, they clustered only with “focus past” and “focus present.” (2) In SFM High and POM High, career-related factors (“power,” “money,” “achieve”) clustered with “risk,” as well as with “positive emotion.”

**Figure 8:**
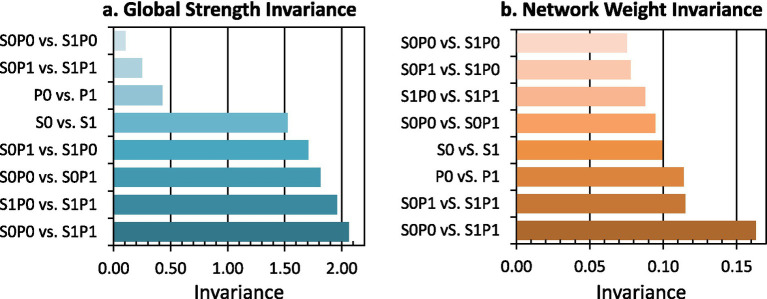
Global strength invariance and network weight invariance across subnetworks. Colors indicate the degree of invariance between pairs of subnetworks. Darker shades denote higher invariance. **(a)** Invariance in global edge strength. **(b)** Invariance in edge weights. S0, low SFM; S1, high SFM; P0, low POM; P1, high POM.

Additionally, we applied the network comparison test to evaluate differences among the eight MIL subnetworks. We summarized each network’s overall edge strength and node proximity using global strength and network weight, and we compared these two indices pairwise across subnetworks. As shown in Figure 8, the largest differences occurred between the SFM Low and POM Low and SFM High and POM High subnetworks, whereas the smallest differences occurred between the SFM Low and POM Low and SFM High and POM Low subnetworks. These patterns suggested that POM exerted a stronger organizing influence on the MIL-related factor structure than SFM. When POM was low, changing SFM produced minimal structural change, while networks diverged most when both POM and SFM shifted from low to high.

## 4 Discussion

This study constructed three-stage deep learning MIL assessment models and extracted associated factors from MIL-related microblogs. The results aligned with Steger’s three-dimensional model (20), which included coherence, purpose, and significance. *Coherence* referred to making sense of one’s life and integrating experiences over time and context. *Purpose* referred to having valued goals and a sense of direction. *Significance* referred to perceiving one’s life as worthwhile and important. (1) Our findings on temporal and spatial components reflected the coherence dimension. Under Low POM, “space” and “time” clustered with “focus future” only in the High SFM and Low POM condition, not in Low SFM and Low POM (see Supplementary Figure F3). This pattern was consistent with the perspective that individuals high in search but lower in presence tend to look ahead and evaluate meaning using future-oriented criteria (49). (2) Results on purpose-related factors were evidenced in two ways. We operationalized purpose-related factors as including “work,” “social,” “achieve,” “power,” “money,” and “risk,” based on the LIWC/associated-factor mapping (Supplementary Table D1). This set was indicative rather than exhaustive, and other categories might also reflect purpose depending on context. First, for both “social” and “work,” centrality was highest in Low SFM and High POM (Supplementary Table F1). Second, career-related factors (“power,” “money,” “achieve”) clustered with risk in the High SFM and High POM subnetwork (Supplementary Figure F3). (3) For the significance dimension, “positive emotion,” “religion,” and “achieve” jointly served as indicative cues of perceived significance. Supplementary Table F1 showed that “positive emotion” and “religion” exhibited the highest centrality in High SFM and High POM, and “achieve” was highest in High SFM contexts. The preliminary convergence between the two-dimensional and three-dimensional MIL frameworks was exploratory rather than confirmatory. Further investigation was needed to provide definitive evidence.

According to the results reported in section 3.2.2 and Figure 5, corpus-level tendencies could be read through Self-Determination Theory (3). Language emphasizing everyday activities in higher POM and lower SFM groups might have indicated a greater focus on autonomy-related routines, whereas the higher use of second person forms in lower POM and lower SFM groups may have reflected a stronger orientation to social-relatedness in this context. The prominence of interrogation terms in High SFM posts was consistent with work connecting exploratory tendencies with active information seeking (50). In addition, prior research reported a negative association between POM and death anxiety (11). While LIWC “Death” reflected factor frequency rather than anxiety, the higher rates of death-related factors in High POM groups might be compatible with an interpretation that individuals with greater POM could approach mortality themes with less anxiety (i.e., more open, approach-oriented processing), though this remains speculative. These interpretations should be treated as tendencies within this corpus and might depend on platform and cultural conventions rather than stable person-level traits.

The centrality patterns reported in section 3.3.1 offered a coherent picture of how different factors may shape MIL (see Supplementary Table F1). The prominence of “attitude,” “emotion,” and “inner thoughts” at the category level suggested that evaluative stance, affective tone, and reflective cognition were central to meaning construction. The high centrality of “biology” was consistent with accounts linking physical condition to MIL, including associations with lower pain, anxiety, and depression, better illness acceptance, and improved quality of life [e.g., (51–53)]. Likewise, the relatively high values for “social,” “work,” and “leisure” aligned with evidence that social roles and support are positively related to MIL (54). In addition, subnetwork-specific patterns reported were consistent with prior theorizing. Positive emotion showed a lower rank under Low POM. By contrast, it showed a higher rank under High POM and High SFM. This pattern was consistent with the view that diminished POM was accompanied by attempts to reduce negative affect. Meanwhile, higher MIL was associated with cultivating positive affective states (7). The greater centrality of “compare” in Low POM subnetworks echoed research on social comparison as a means of status appraisal under uncertainty (55). The higher centrality of “shehe” in Low POM and High SFM might reflect an outward orientation toward models or referents when search was high but presence was limited (56).

Building on the correlation patterns reported in section 3.3.2 (Supplementary Figures F1, F2; Supplementary Tables F2, F3), we interpreted three descriptive regularities. First, pairs involving “space”/“time” with “relative” recurred among the higher correlations across contexts. This was consistent with the idea that appraisals of change and continuity drew on temporal and spatial comparisons (22). Second, within High POM, the association between “body” and “bio” was among the higher pairs. This aligned with the view that connected bodily states and recovery experiences were linked to meaning. Prior work linked meaning to pain experiences and related higher POM to lower health anxiety (57, 58). Third, within Low POM, the association between “power” and “drive” was also among the higher pairs. This fit perspectives that lower POM could co-occur with compensatory striving (59). These interpretations were intended to contextualize the observed correlations and remained descriptive rather than inferential. No causal claims were intended.

Building on the structural patterns reported in section 3.3.3 (Supplementary Figure F3), we offered two descriptive interpretations. First, under High POM and High SFM, the alignment of risk with career-related factors and positive emotion fit perspectives that more satisfied individuals pursued new achievements even in the face of risk (60). Second, “we” clustered with “temporal” “spatial” and “comparative” factors in Low SFM and Low POM, but with “social” factors in High POM and Low SFM. This pattern was compatible with the view that higher presence related to finding meaning in social roles and positive interactions (61). These interpretations were descriptive rather than inferential.

This study adopted a dual data collection strategy, combining a large-scale corpus of over 3 million publicly available microblogs with a smaller participant dataset that integrated both self-report questionnaires and personal microblogs. The large-scale dataset enabled population-level analysis of MIL, while the participant dataset provided validated ground truth by linking subjective measures to corresponding online expressions. These complementary datasets allowed model validation from three perspectives: human cross-annotation, ChatGPT-based labeling, and user self-report surveys. Each method carried distinct strengths and limitations. Human annotation, often considered the gold standard, offered nuanced interpretations but was resource-intensive and subject to inter-annotator variability. ChatGPT-based labeling was used as a supplemental convergent check of human annotation, as it provided scalability and efficiency (62). However, the outputs of ChatGPT were sensitive to prompts and sometimes prone to hallucination (63). In addition, ChatGPT models pretrained on general corpora had difficulty in accurately identifying and evaluating the construct ambiguity of MIL, as the boundaries between POM and SFM were sometimes overlapping. Therefore, although the judgments of ChatGPT-based validation were largely consistent in direction with human annotations, the overall accuracy was lower. User surveys grounded predictions in participants’ self-reports and enhanced ecological validity, though they remained vulnerable to recall bias, social desirability, and limited sample size. Taken together, the convergences and divergences across these approaches underscored the value of triangulation: manual coding secured high-quality benchmarks, GPT-assisted annotation enabled efficient large-scale analysis, and user self-report anchored computational predictions to psychological ground truth. Future work may benefit from hybrid strategies that combine LLM-assisted pre-annotation with human oversight, alongside triangulation using self-reported measures (64).

The performance of our proposed MIL assessment models on the large-scale dataset varied substantially. Model 1 identified whether a post was related to MIL, while Models 2 and 3 classified the two dimensions of MIL, namely POM and SFM. As dimensions of MIL, POM and SFM were conceptually more complex and theoretically debated. For instance, previous debates have concerned whether the two dimensions overlap or should be separated, as proposed in three-dimensional models (19, 20). This conceptual ambiguity increased task difficulty. Moreover, both dimensions represented latent psychological constructs that were expressed more implicitly in social media text, making them more challenging for both human annotation and model classification. During training data construction, the inter-annotator agreement (Cohen’s kappa = 0.84 for MIL, 0.71 for SFM, and 0.68 for POM; all *p* < 0.01; see section 2.1.1) reflected this trend. Therefore, the pattern in which Model 1 (≈90%) outperformed Models 2 and 3 (≈75%) was consistent with human annotation reliability. As these estimates were based on a sampled subset, they should be interpreted as exploratory references rather than definitive conclusions.

This study had limitations. First, we did not differentiate by demographics. Because MIL could arise from different sources at different life stages, categorizing participants could facilitate understanding of associated factors within groups. Second, our MIL assessment models focused on the post level rather than on users’ posts over time. Assessing MIL at the user level could address the sparsity of microblog data and add temporal cues, potentially improving model performance. Third, in the multi-level network analysis of MIL-associated factors, no inferential statistics were performed. All statements were descriptive rather than inferential and were not presented as evidence of statistical significance or causality. Future studies will test these differences with formal inferential methods. Fourth, the representativeness and cultural generalizability of our findings were limited. Our text corpus consisted of posts written in Chinese and posted at night on the Sina Weibo platform. Because culture and platform norms may have shaped language use, expressions of MIL associated factors might differ on other social media platforms and in other countries. In addition, the participants in the empirical study were demographically specific (see Supplementary Table E3). For example, 69.9% were under 24 years old and 72.3% were undergraduates, master’s, or PhD students. Socioeconomic status was not collected. Therefore, replication across platforms, languages, cultures, and demographics was needed before broader generalization. In addition, future work could assess MIL in non-sleep contexts to enable comparative analyses and further examine generalization.

Three future research directions were envisioned. First, we will examine the relationship between perceived social support in social networks and users’ MIL. Interpersonal relationships are expected to be a significant source of meaning and a predictor of MIL (65). Clues related to “social” and “friend” were identified in this study, indicating that the category “we” clustered with social categories only under the condition of High SFM and High POM. Second, images in social networks, which contain rich visual information reflecting users’ interests, values, and emotional states (66), will be investigated to determine whether visual information can improve the precision of MIL assessment. Third, this study provided a preliminary exploration of using prompts based on a pretrained large language model to label components and levels of MIL, but the performance was limited. Future studies may attempt fine-tuning large language models on manually annotated MIL corpora to further improve both efficiency and accuracy of automatic MIL assessment.

## 5 Conclusion

Using social media text, we proposed a structured three-part framework for assessing MIL along SFM and POM and validated the models with multiple approaches. Through graph-based semantic analysis, we identified associated factors from MIL expressions. We then explored their large-scale patterns across groups. These findings outline context-dependent topic and network differences associated with MIL levels and provided a data-driven lens for monitoring MIL-related signals in online populations. While exploratory and descriptive, this work may help prioritize factors for follow-up assessment and inform the design of supportive, evidence-guided interventions.

## Data Availability

The datasets presented in this study can be found in online repositories. The names of the repository/repositories and accession number(s) can be found in the article/Supplementary material.
