# Supplementary material for: Exploring meaning in life from social network content in the sleep scenario
Source: Front Public Health. 2025 Nov 11;13:1642085. doi: 10.3389/fpubh.2025.1642085 (PMC12643844; doi:10.3389/fpubh.2025.1642085)
Supplement: Supplementary file 1 [file Data_Sheet_1.pdf]

Appendix A. Examples of Stop Words

Table A1. *Stopword List: Examples by Category*

	Category	Example 1	Example 2	Quantity
1	Studying	MoMo Vocabulary	#Shanbei Check-in#	10
2	Lottery	Lucky Red Envelope	1 Yuan Flash Sale	132
3	Fan Engagement	#Celebrity Power Rankings#	#EXO	146
4	Sports Events	Winter Olympics Badge	Eileen Gu	47
5	Film & TV	New Year's Eve Gala	#Sina Movie Watching Group#	30
6	Gaming	#Weibo Game Awards#	#Jianwang 3	72
7	Advertising	Credamo	#Sina Weipan iOS Client#	5
8	Environmental	#Panda Guardians#	Idol Environmental Energy	6
9	Check-ins	Visible Meals	#Weibo Video Check-in Plan#	16
10	Sharing	#Spring Atmosphere Special Effects#	#What Did Sister Buy#	63

Note. The stopword list comprises 10 categories and 469 words. Some words belong to multiple categories, and some categories are superordinate topics. The dataset and code are available at: https://pan.bnu.edu.cn/l/p1Cjcs.

Appendix B. Guidelines for Annotating MIL in Posts

Background: Welcome to the annotation work of our research team! The goal of our current work is to use natural language processing models to automatically assess the meaning in life (MIL) reflected in Weibo texts. Your task is to annotate posts. Accurate annotation is very important for the effectiveness of our subsequent model training. Please read each post carefully and strictly follow the annotation instructions below.

(1) **Task 1:** Determine if the post is related to MIL.
  (a) If you believe the post expresses MIL, label it as "Related."
  (b) If you think the post is not related to MIL, label it as "Not Related" and do not proceed to the next step.
  (c) If you find it difficult to make a judgment, label it as "Unable to Judge."
  (d) Additionally, if the content of the post is an advertisement, a novel excerpt, etc., label it as "Not Related," even if it involves the concept of meaning in life.

(2) **Task 2:** For MIL-related posts, further label the "Presence of Meaning (POM)" dimension.
  (a) If you think the post expresses a strong sense of presence of meaning in life, label it as "High POM." Otherwise, label it as "Low POM."
  (b) If you find it difficult to make a judgment, label it as "Unable to Judge."

(3) **Task 3:** For MIL-related posts, further label the "Search for Meaning (SFM)" dimension.
  (a) If you think the post expresses a strong sense of searching for meaning in life, label it as "High SFM." If not, label it as "Low SFM."
  (b) If you find it difficult to make a judgment, label it as "Unable to Judge."

We adopt cross-annotation for each task, meaning that each post will be independently labeled by two annotators. Only posts labeled as MIL-related in Task 1 need to be further labeled for Task 2 and Task 3. After all annotations are complete, the

research team will assess annotation consistency, and payment will be based on the level of agreement. The rules for determining the consistency of the annotation results are shown in Table B1. Consistency for each task is calculated separately. Table B2 are some examples to further explain the annotation tasks.

Table B1. Example of the Rules for Assessing Annotation Consistency ('-' Indicates 'No Judgment').

Post	Task	Annotator1	Annotator2	If consistent
Post1	Task1 (If related)	Related	Unrelated	No
	Task2 (POM)	Related, High POM	-	No
	Task3 (SFM)	Related, Low SFM	-	No
Post2	Task1 (If related)	Related	Related	Yes
	Task2 (POM)	Related, High POM	Related, High POM	Yes
	Task3 (SFM)	Related, Low SFM	Related, High SFM	No
Post3	Task1 (If related)	Unrelated	Unrelated	Yes
	Task2 (POM)	-	-	-
	Task3 (SFM)	-	-	-
Post4	Task1 (If related)	Related	Related	Yes
	Task2 (POM)	Unable to Judge	Related, Low POM	No
	Task3 (SFM)	Related, Low SFM	Related, Low SFM	Yes

Table B2. Examples for MIL post annotation.

ID	Post	Annotations						
		MIL-related or not	SFM			POM		
			High	Low	Unable To Judge	High	Low	Unable To Judge
1	Life advice: Don't sleep on the couch.	Unrelated						
2	I really like this saying: In life, you should blaze your own trail through the mountains and build bridges over the water.	Related	✓				✓	
3	Recent life: Overeating, excessive late nights. Oops! Change it!	Related	✓					✓
4	Live to be happy, happy laugh, tired sleep, life is not necessarily cool, but must have an attitude.	Related		✓		✓		
5	The stress of life wears me out.	Related		✓			✓	

Appendix C. Pseudocode for Algorithms

Table C1

Pseudocode for Constructing Semantic Dependency Graphs and Identifying Root Causes

Description

Input: Post p ∈ P, LTP pipeline, role set 𝓡, max depth L = 3

Output: Graph G = (V, E), Root causes 𝓒

```
1  Sentences ← LTP.SentenceSplit(p)
2  Tokens, POS, Dep ← LTP.TokenizeTagParse(Sentences)
3  Units ← MapToSemanticUnits(Tokens, POS, Dep)
4  V ← ∅ ; E ← ∅
5  Seeds ← { (uA, uB) | ((uA, uB), REAS) ∈ Units }
6  for each (uA, uB) in Seeds do
7     V ← V ∪ {uA, uB} ; E ← E ∪ {((uA, uB), REAS)}
8  Set ← Seeds ; ℓ ← 1
9  while Set ≠ ∅ and ℓ ≤ L do
10    Next ← ∅
11    for each pair (x, y) in Set do
12       Neigh ← NeighborByRoles(x, y, Units, 𝓡)
13       for each ((a, b), r) in Neigh do
14          V ← V ∪ {a, b} ; E ← E ∪ {((a, b), r)}
15          Next ← Next ∪ {(a, b)}
16    Set ← Next ; ℓ ← ℓ + 1
17 G ← (V, E)
18 score(u) ← Degree(u in G) + α · SignedStrength(u)
19 𝓒 ← TopK({u ∈ V | incident to REAS-paths}, by score)
20 return G, 𝓒
```

Note.

Inputs: posts P; LTP = Language Technology Platform pipeline; role set 𝓡 = {REAS = reason, AGT = agent, EXP = experiencer, PAT = patient/object, CONT = content, DATV = dative, LINK = link, TIME = temporal, LOC = locative}.

Output: semantic dependency graph G and root-cause set 𝓒.

Step objectives. Steps 1–3 segment text and extract semantic role tuples; 4–17 expand the dependency graph from REAS up to L layers; 18–20 score candidate root causes by connectivity and strength.

Table C2

Pseudocode for Event-Component Extraction and LIWC Mapping

Description
**Input:** Graph G = (V, E), stopwords $\boldsymbol{\mathcal{S}}$, LIWC lexicon, consolidation map φ
**Output:** $\boldsymbol{\mathcal{K}}$ (keywords), $\boldsymbol{\mathcal{M}}$ (LIWC category counts), $\boldsymbol{\mathcal{G}}$ (9-group counts)
1 Candidates ← ∅
2 for each edge ((a, b), r) ∈ E do
3   if r ∈ {REAS, AGT, EXP, PAT, CONT, DATV, LINK, TIME, LOC} then
4   Candidates ← Candidates ∪ {a, b}
5 $\boldsymbol{\mathcal{K}}$ ← NormalizeFilter(Candidates, stopwords = $\boldsymbol{\mathcal{S}}$)
6 $\boldsymbol{\mathcal{M}}$ ← ZeroCounts over 52 LIWC categories
7 for each term t ∈ $\boldsymbol{\mathcal{K}}$ do
8   C ← MatchLIWC(t)
9   for each c ∈ C do
10       $\boldsymbol{\mathcal{M}}$[c] ← $\boldsymbol{\mathcal{M}}$[c] + 1
11 $\boldsymbol{\mathcal{G}}$ ← ZeroCounts over 9 consolidated groups
12 for each LIWC category c do
13   g ← φ(c)
14    $\boldsymbol{\mathcal{G}}$[g] ← $\boldsymbol{\mathcal{G}}$[g] + $\boldsymbol{\mathcal{M}}$[c]
15 return $\boldsymbol{\mathcal{K}}$, $\boldsymbol{\mathcal{M}}$, $\boldsymbol{\mathcal{G}}$

Note.

Inputs: G = semantic dependency graph; $\boldsymbol{\mathcal{S}}$ = stopword list; LIWC lexicon = Chinese LIWC (52 categories); φ = mapping from 52 categories to 9 groups (Table C1).

Output: $\boldsymbol{\mathcal{K}}$ = event-component keywords, $\boldsymbol{\mathcal{M}}$ = LIWC category counts, $\boldsymbol{\mathcal{G}}$ = consolidated group counts..

Step objectives. Steps 1–4 collect candidate terms from graph edges; 5 normalize and remove stopwords; 7–10 map terms to LIWC categories; 11–14 consolidate into 9 broader groups (see Table C1).

Table C3

List of Variables and Symbols

Description
· P: set of posts; p: individual post
· $\mathcal{R}$: role set {REAS, AGT, EXP, PAT, CONT, DATV, LINK, TIME, LOC}
· L: maximum expansion depth (3 in this study)
· G = (V, E): semantic dependency graph, with node set V and edge set E
· $\mathcal{C}$: root cause set (top-k nodes ranked by score)
· $\mathcal{S}$: stop word list
· $\mathcal{K}$: event-component keyword set
· $\mathcal{M}$: counts of LIWC categories (52)
· $\mathcal{G}$: counts of consolidated LIWC groups (9)
· φ: mapping from 52 LIWC categories to 9 consolidated groups
· Degree(u): degree of node u
· SignedStrength(u): signed strength (positive/negative) of edges for node u
· α: weight parameter (≥ 0)

Note. Variables and symbols used in Tables D1 and D2.

Appendix D. LIWC Categories and Groups Utilized in this Study

Table D1. LIWC Categories Adopted in This Study

Group	Categories in LIWC
Attitude (4)	Negate (e.g., no, don't)
	Compare (e.g., different, decline)
	Relative (e.g., relative, compare)
	Assent (e.g., agree, good)
Context (4)	See (e.g., look, green)
	Hear (e.g., hear, yell)
	Space (e.g., street, home)
	Time (e.g., Autumn, during)
Emotion (7)	Positive emotion (e.g., confident, sastified)
	Negative emotion (worry, suspect)
	Anxiety (struggle, uptight)
	Anger (e.g., hateful, complain)
	Sad (e.g., heartburn, dispirited)
	Drive (e.g., fear, opinion)
	Swear (e.g., silly)
Gender groups (2)	Female (e.g., maternity, daughter)
	Male (e.g., father, prince)
Inner thoughts (10)	Insight (e.g., understand, realize)
	Cause (e.g., reason, cause)
	Discrepancy (e.g., wonder, lack, expect)
	Certain (e.g., definitely, sure)
	Consciousness (e.g., warm, experience)
	Feel (e.g., smoothness, touch)
	Achieve (e.g., be good at, master-hand)
	Power (e.g., justice, permit)
	Reward (e.g., score, brave)
	Risk (e.g., loss, suspend)
Interrogatioin (1)	Interrogatioin (e.g.,when, what)
Personal pronoun (6)	First person singular (e.g., I, in person)
	First person plural (e.g., we, both of us)
	Second person singular (e.g., you)
	Third person singular (e.g., he, she)
	Third person plural (e.g., they)
	Second person plural (you)
Tense (4)	Past tense (e.g., last year, just now)
	Present tense (e.g., now, usually)
	Future tense (e.g., after, future)
	Progressive tense (e.g., so far, recently,)
factor (14)	Social (e.g., adopt, greet)
	Family (e.g., brother, parents-in-law)
	Friend (e.g., companion, friend)
	Biology (e.g., sweat, hug)
	Body (e.g., neck, skin)
	Health (e.g., insomnia, doctor)
	Sexual (e.g., sex, naked)
	Ingest (e.g., eat, cook)
	Work (e.g., factory, interview)
	Leisure (e.g., sing, holiday)
	Home (e.g., house, pet)
	Money (e.g., rich, salary)
	Religion (e.g., god, belief)
	Death (e.g., suicide, will)

Note. We selected 52 LIWC categories, grouped into 9 clusters. This dictionary guided our extraction of associated factors from semantic dependency graphs derived from posts about life's meaning.

Appendix E. Prompt Used for ChatGPT's Assessment of MIL

Table E1. We used ChatGPT to automatically evaluate the user's MIL and the associated factors. Specifically, we designed a Python program to use the "gpt-3.5-turbo" model through the ChatGPT API key. Given a user's post, we designed a prompt and sent it to ChatGPT to obtain the evaluated result. This prompt initially provided an example to instruct the model on the task (answering six questions about MIL) and then included a new post to instruct the model to perform the same task.

The prompt for ChatGPT's automatic assessment of life's meaning based on microblog content.
You are a psychologist. Please answer the following questions based on the text posted by a social media user.<br>**Input**:<br>Paragraph:<br>user's microblog content<br>Q1: If this text is relevant to a sense of meaning in life?<br>Q2: If so, what is the level of the "search for meaning" dimension (high/low)?<br>Q3: What is the level of the "having meaning" dimension (high/low)?<br>Q4: If the top-1 factor factor1 is the trigger for the current sense of meaning in life?<br>Q5: If the top-3 factors factor1, factor2, factor3 contain reasons that triggered the current sense of meaning in life?<br>Q6: If the top-5 factors factor1, factor2, factor3, factor4, factor5 contain triggers for the current sense of meaning in life?<br>**Output**:<br>R1: Yes; R2: High; R3: Low; R4: No; R5: Yes; R6: Yes.<br>**Input**:<br>Paragraph:<br>user's microblog content (new)<br>Q1: If this text is relevant to a sense of meaning in life?<br>Q2: If so, what is the level of the "search for meaning" dimension (high/low)?<br>Q3: What is the level of the "having meaning" dimension (high/low)?<br>Q4: If the top-1 factor factor1 is the trigger for the current sense of meaning in life?<br>Q5: If the top-3 factors factor1, factor2, factor3 contain reasons that triggered the current sense of meaning in life?<br>Q6: If the top-5 factors factor1, factor2, factor3, factor4, factor5 contain triggers for the current sense of meaning in life?<br>**Output**:<br>(ChatGPT's Results)

Table E2. Characteristics of Surveyed Users Who Had Posted Microblogs Related to MIL (Assessed by Model 1).

		Full Sample (N = 177)	MIL Score <= 35 (N = 99)	MIL Score > 35 (N = 77)	p-value
Ratio of MIL-related posts		0.10	0.075	0.123	0.006**
Average number of posts		161.7	188.0	129.9	.152 (0.04* if N=289)
Age	18-24	132(75%)	76	56	0.313^a
	25-34	38(21.6%)	21	17	-
	>35	6(3.4%)	2	4	-
Gender	Male	20(11.4%)	8	12	0.12^a
	Female	156(88.6%)	91	65	-
Education	Undergraduate	98(55.7%)	53	45	0.493^a
	Master's or PhD	39(22.6%)	23	16	-
	Employed	38(21.6%)	23	15	-
	Unemployed	1(0.5%)	0	1	-

*$p < 0.05$, **$p < 0.01$, [a]Chi-square test.

Table E3. Demographics of Valid, Active Survey Participants (N = 289)

Characteristic	n	%
**Age**		
< 18	1	0.3%
18–24	208	69.6%
25–34	70	23.4%
> 35	10	3.3%
**Gender**		
Male	63	21.8%
Female	226	78.2%
**Education / Status**		
High school or junior high school students	5	1.7%
Undergraduate student	158	54.7%
Master's or PhD student	51	17.6%
Employed	70	24.6%
Unemployed	4	1.4%

Note. Participants were selected from 923 recruits using two criteria: (1) posted at least 10 valid, original microblog posts; (2) passed the lie-detection item and showed no patterned responding (e.g., selecting the same option for all items).

Appendix F. Supplementary Results

Table F1. Node centrality indices (Strength) of eight MIL subnetworks, ranked by Expected Influence.

Factor	High SFM	Low SFM	High POM	Low POM	Low SFM & Low POM	Low SFM & High POM	High SFM & Low POM	High SFM & High POM	Average
Bio	2.08	2.12	2.08	2.14	2.13	2.02	2.06	2.04	2.08
Percept	1.73	1.79	1.74	1.82	1.79	1.80	1.74	1.72	1.77
Drives	1.64	1.58	1.58	1.60	1.58	1.63	1.56	1.62	1.60
Negemo	1.48	1.46	1.49	1.46	1.45	1.50	1.49	1.49	1.48
Social	1.34	1.34	1.35	1.35	1.34	1.37	1.34	1.34	1.35
Insight	1.21	1.20	1.21	1.19	1.20	1.16	1.19	1.21	1.20
Relativ	1.15	1.15	1.15	1.16	1.16	1.17	1.15	1.14	1.15
Posemo	1.16	1.17	1.15	1.14	1.16	1.08	1.09	1.20	1.14
Time	1.12	1.16	1.14	1.15	1.16	1.14	1.14	1.13	1.14
Shehe	1.10	1.10	1.08	1.06	1.05	1.07	1.13	1.04	1.08
Assent	1.05	1.08	1.07	1.08	1.09	1.05	1.08	1.04	1.07
Space	1.00	1.02	1.01	1.01	1.02	1.01	1.00	1.00	1.01
Discrep	0.92	0.97	0.96	0.95	0.96	0.93	0.99	0.93	0.95
Cause	0.96	0.93	0.97	0.92	0.90	0.93	0.95	0.98	0.94
Compare	0.90	0.95	0.92	0.96	0.95	0.91	0.93	0.92	0.93
Certain	0.84	0.98	0.89	0.97	1.00	0.92	1.00	0.81	0.93
Progm	0.92	0.89	0.90	0.89	0.88	0.92	0.89	0.93	0.90
Focusfuture	0.88	0.87	0.89	0.86	0.85	0.87	0.93	0.89	0.88
I	0.86	0.88	0.86	0.85	0.89	0.83	0.88	0.86	0.86
Male	0.87	0.83	0.86	0.87	0.85	0.92	0.82	0.86	0.86
Power	0.82	0.86	0.89	0.84	0.83	0.80	0.92	0.86	0.85
Negate	0.85	0.85	0.83	0.88	0.85	0.86	0.83	0.82	0.85
Work	0.82	0.86	0.84	0.87	0.86	0.88	0.85	0.77	0.84
Anger	0.89	0.74	0.86	0.81	0.80	0.91	0.73	0.94	0.83
Risk	0.84	0.80	0.87	0.78	0.76	0.83	0.89	0.86	0.83
Achieve	0.82	0.79	0.81	0.76	0.79	0.72	0.81	0.81	0.79
Focuspresent	0.77	0.75	0.78	0.74	0.74	0.73	0.76	0.80	0.76
Female	0.76	0.71	0.74	0.75	0.76	0.73	0.73	0.77	0.74
Leisure	0.68	0.74	0.70	0.76	0.75	0.80	0.76	0.66	0.73
Focuspast	0.71	0.70	0.69	0.71	0.71	0.73	0.69	0.73	0.71
Interrog	0.75	0.65	0.73	0.63	0.64	0.68	0.71	0.79	0.70
Family	0.68	0.64	0.68	0.62	0.69	0.59	0.61	0.71	0.65
You	0.64	0.66	0.63	0.67	0.66	0.67	0.64	0.63	0.65
Reward	0.54	0.62	0.57	0.62	0.64	0.61	0.67	0.53	0.60
Hear	0.64	0.53	0.62	0.53	0.54	0.53	0.56	0.68	0.58
Body	0.63	0.51	0.60	0.50	0.48	0.58	0.58	0.66	0.57
Sad	0.51	0.61	0.55	0.59	0.62	0.47	0.54	0.55	0.55
Home	0.51	0.58	0.49	0.59	0.58	0.65	0.56	0.45	0.55
Feel	0.60	0.48	0.54	0.49	0.50	0.52	0.45	0.56	0.52
See	0.56	0.48	0.53	0.47	0.49	0.46	0.49	0.54	0.50
Swear	0.50	0.46	0.49	0.49	0.48	0.56	0.46	0.46	0.49
Anx	0.34	0.51	0.38	0.47	0.52	0.38	0.49	0.35	0.43
Money	0.42	0.43	0.43	0.41	0.42	0.47	0.43	0.41	0.43
Health	0.45	0.34	0.44	0.35	0.35	0.48	0.40	0.48	0.41
We	0.39	0.39	0.36	0.41	0.45	0.36	0.34	0.39	0.39
Friend	0.40	0.35	0.34	0.38	0.37	0.46	0.40	0.36	0.38
Sexual	0.43	0.30	0.40	0.29	0.26	0.47	0.41	0.42	0.38
Relig	0.37	0.31	0.38	0.30	0.33	0.28	0.34	0.41	0.34
Death	0.31	0.33	0.27	0.29	0.34	0.28	0.31	0.35	0.31
They	0.35	0.24	0.31	0.24	0.26	0.33	0.28	0.33	0.29
Youpl	0.30	0.24	0.26	0.28	0.28	0.36	0.27	0.22	0.28
Ingest	0.17	0.20	0.24	0.15	0.24	0.22	0.29	0.20	0.22

Note. Each node corresponds to one of the 52 LIWC categories. Higher node-centrality values indicates greater influence. For example, in the High SFM & Low POM subnetwork, the top nodes include “bio”, “perception”, and “drive”.

Table F2. Top 15 correlations among the node-to-node relations of MIL sub-networks.

		Assent	Bio	Body	Cause	Compare	Drives	Insight	Interrog	Male	Negate	Negemo	Power	Progm	Relativ	Shehe	Social	Space	Time
S0P1	Shehe	0.378	0.302	0.240	0.342	0.369	0.377	0.382	0.303	**0.770**	0.349	0.353	0.320	0.366	0.382	1.000	0.481	0.381	0.375
	Negate	0.613	0.529	0.431	0.499	0.595	0.648	0.606	0.436	0.309	1.000	0.818	0.518	0.563	0.644	0.349	0.617	0.616	0.591
	Progm	**0.872**	0.558	0.450	0.528	0.655	0.645	**0.903**	0.417	0.336	0.563	0.592	0.524	1.000	0.730	0.366	0.648	0.661	0.665
	Compare	0.711	0.570	0.450	0.542	1.000	0.681	0.697	0.436	0.336	0.595	0.615	0.550	0.655	0.750	0.369	0.680	0.715	0.706
	Negemo	0.644	0.593	0.476	0.524	0.615	0.668	0.638	0.446	0.317	**0.818**	1.000	0.555	0.592	0.676	0.353	0.640	0.638	0.616
	Social	0.718	0.569	0.438	0.564	0.680	**0.758**	0.711	0.473	0.439	0.617	0.640	0.597	0.648	**0.755**	0.481	1.000	0.717	0.684
	Male	0.349	0.278	0.224	0.311	0.336	0.342	0.349	0.277	1.000	0.309	0.317	0.292	0.336	0.342	0.770	0.439	0.346	0.346
	Insight	**0.873**	0.586	0.466	0.580	0.697	0.703	1.000	0.463	0.349	0.606	0.638	0.562	0.903	0.771	0.382	0.711	0.705	0.688
	Bio	0.594	1.000	0.779	0.444	0.570	0.591	0.586	0.368	0.278	0.529	0.593	0.489	0.558	0.617	0.302	0.569	0.583	0.584
	Body	0.469	**0.779**	1.000	0.362	0.450	0.454	0.466	0.299	0.224	0.431	0.476	0.390	0.450	0.489	0.240	0.438	0.467	0.470
	Drives	0.722	0.591	0.454	0.564	0.681	1.000	0.703	0.437	0.342	0.648	0.668	0.769	0.645	0.768	0.377	0.758	0.744	0.677
	Power	0.563	0.489	0.390	0.463	0.550	**0.769**	0.562	0.354	0.292	0.518	0.555	1.000	0.524	0.620	0.320	0.597	0.641	0.543
	Relativ	**0.790**	0.617	0.489	0.593	**0.750**	**0.768**	**0.771**	0.471	0.342	0.644	0.676	0.620	0.730	1.000	0.382	0.755	**0.883**	**0.821**
	Space	0.717	0.583	0.467	0.546	0.715	0.744	0.705	0.435	0.346	0.616	0.638	0.641	0.661	0.883	0.381	0.717	1.000	0.715
	Time	0.694	0.584	0.470	0.547	0.706	0.677	0.688	0.433	0.346	0.591	0.616	0.543	0.665	0.821	0.375	0.684	0.715	1.000
	Assent	1.000	0.594	0.469	0.567	0.711	0.722	0.873	0.459	0.349	0.613	0.644	0.563	0.872	0.790	0.378	0.718	0.717	0.694
S0P0	Shehe	0.371	0.303	0.250	0.331	0.350	0.361	0.379	0.270	**0.786**	0.339	0.339	0.310	0.361	0.365	1.000	0.460	0.362	0.354
	Negate	0.600	0.504	0.399	0.488	0.594	0.631	0.595	0.398	0.310	1.000	0.808	0.517	0.545	0.627	0.339	0.614	0.595	0.591
	Progm	**0.856**	0.505	0.404	0.510	0.635	0.639	**0.885**	0.389	0.324	0.545	0.578	0.521	1.000	0.705	0.361	0.651	0.659	0.657
	Compare	0.699	0.532	0.416	0.530	1.000	0.688	0.688	0.418	0.320	0.594	0.614	0.563	0.635	0.749	0.350	0.706	0.724	0.724
	Negemo	0.636	0.572	0.439	0.512	0.614	0.668	0.632	0.412	0.307	**0.808**	1.000	0.550	0.578	0.668	0.339	0.654	0.623	0.617
	Social	0.723	0.563	0.420	0.569	0.706	**0.756**	0.730	0.450	0.416	0.614	0.654	0.589	0.651	**0.772**	0.460	1.000	0.726	0.699
	Male	0.337	0.279	0.237	0.291	0.320	0.335	0.341	0.249	1.000	0.310	0.307	0.284	0.324	0.333	0.786	0.416	0.330	0.325
	Insight	**0.855**	0.547	0.432	0.565	0.688	0.704	1.000	0.435	0.341	0.595	0.632	0.564	0.885	0.763	0.379	0.730	0.711	0.693
	Bio	0.561	1.000	0.748	0.421	0.532	0.580	0.547	0.333	0.279	0.504	0.572	0.488	0.505	0.587	0.303	0.563	0.556	0.554
	Body	0.437	**0.748**	1.000	0.333	0.416	0.433	0.432	0.257	0.237	0.399	0.439	0.384	0.404	0.442	0.250	0.420	0.431	0.431
	Drives	0.724	0.580	0.433	0.549	0.688	1.000	0.704	0.405	0.335	0.631	0.668	0.783	0.639	0.776	0.361	0.756	0.748	0.697
	Power	0.577	0.488	0.384	0.459	0.563	**0.783**	0.564	0.341	0.284	0.517	0.550	1.000	0.521	0.628	0.310	0.589	0.641	0.559
	Relativ	**0.776**	0.587	0.442	0.574	**0.749**	**0.776**	**0.763**	0.442	0.333	0.627	0.668	0.628	0.705	1.000	0.365	0.772	0.895	0.827
	Space	0.721	0.556	0.431	0.538	0.724	0.748	0.711	0.413	0.330	0.595	0.623	0.641	0.659	**0.895**	0.362	0.726	1.000	0.733
	Time	0.700	0.554	0.431	0.534	0.724	0.697	0.693	0.403	0.325	0.591	0.617	0.559	0.657	**0.827**	0.354	0.699	0.733	1.000
	Assent	1.000	0.561	0.437	0.558	0.699	0.724	0.855	0.433	0.337	0.600	0.636	0.577	0.856	0.776	0.371	0.723	0.721	0.700

Note. All correlation coefficients are significant at $p < .001$. 'S0P1' refers to Low SFM and High POM. 'S0P0' refers to Low SFM and Low POM. The 15 strongest correlations between categories are shown in boldface, corresponding to thicker edges and shorter inter-node distances in Figures F1.

Table F3. Top 15 correlations among the node-to-node relations of MIL sub-networks.

		Assent	Bio	Body	Cause	Compare	Drives	Insight	Interrog	Male	Negate	Negemo	Power	Progm	Relativ	Shehe	Social	Space	Time
S1P1	Shehe	0.340	0.270	0.229	0.302	0.328	0.329	0.349	0.273	**0.773**	0.315	0.317	0.282	0.330	0.334	1.000	0.447	0.332	0.324
	Negate	0.621	0.536	0.458	0.506	0.602	0.636	0.609	0.468	0.288	1.000	0.831	0.510	0.562	0.657	0.315	0.601	0.616	0.594
	Progm	**0.853**	0.528	0.453	0.513	0.626	0.611	**0.893**	0.458	0.302	0.562	0.594	0.499	1.000	0.691	0.330	0.615	0.635	0.629
	Compare	0.681	0.551	0.459	0.525	1.000	0.648	0.670	0.473	0.296	0.602	0.631	0.528	0.626	0.730	0.328	0.657	0.689	0.687
	Interrog	0.516	0.405	0.336	**0.735**	0.473	0.470	0.500	1.000	0.252	0.468	0.479	0.377	0.458	0.522	0.273	0.506	0.478	0.456
	Negemo	0.653	0.616	0.524	0.530	0.631	0.662	0.644	0.479	0.292	**0.831**	1.000	0.541	0.594	0.690	0.317	0.629	0.641	0.614
	Social	0.679	0.545	0.442	0.558	0.657	0.699	0.682	0.506	0.409	0.601	0.629	0.556	0.615	**0.712**	0.447	1.000	0.678	0.638
	Male	0.311	0.254	0.208	0.268	0.296	0.305	0.317	0.252	1.000	0.288	0.292	0.257	0.302	0.306	0.773	0.409	0.300	0.297
	Insight	**0.859**	0.571	0.487	0.568	0.670	0.670	1.000	0.500	0.317	0.609	0.644	0.532	0.893	0.743	0.349	0.682	0.679	0.659
	Cause	0.568	0.460	0.391	1.000	0.525	0.537	0.568	0.735	0.268	0.506	0.530	0.441	0.513	0.589	0.302	0.558	0.541	0.531
	Bio	0.579	1.000	0.810	0.460	0.551	0.566	0.571	0.405	0.254	0.536	0.616	0.470	0.528	0.602	0.270	0.545	0.558	0.557
	Body	0.484	**0.810**	1.000	0.391	0.459	0.466	0.487	0.336	0.208	0.458	0.524	0.394	0.453	0.496	0.229	0.442	0.467	0.468
	Drives	0.690	0.566	0.466	0.537	0.648	1.000	0.670	0.470	0.305	0.636	0.662	0.772	0.611	0.732	0.329	0.699	0.711	0.643
	Power	0.537	0.470	0.394	0.441	0.528	**0.772**	0.532	0.377	0.257	0.510	0.541	1.000	0.499	0.592	0.282	0.556	0.618	0.525
	Relativ	**0.763**	0.602	0.496	0.589	**0.730**	**0.732**	**0.743**	0.522	0.306	0.657	0.690	0.592	0.691	1.000	0.334	0.712	0.868	0.811
	Space	0.695	0.558	0.467	0.541	0.689	0.711	0.679	0.478	0.300	0.616	0.641	0.618	0.635	**0.868**	0.332	0.678	1.000	0.681
	Time	0.666	0.557	0.468	0.531	0.687	0.643	0.659	0.456	0.297	0.594	0.614	0.525	0.629	**0.811**	0.324	0.638	0.681	1.000
	Assent	1.000	0.579	0.484	0.568	0.681	0.690	0.859	0.516	0.311	0.621	0.653	0.537	0.853	0.763	0.340	0.679	0.695	0.666
S1P0	Shehe	0.372	0.320	0.278	0.319	0.353	0.364	0.383	0.281	**0.790**	0.334	0.343	0.317	0.365	0.368	1.000	0.456	0.364	0.359
	Negate	0.635	0.537	0.436	0.529	0.634	0.664	0.634	0.450	0.308	1.000	0.827	0.553	0.576	0.671	0.334	0.652	0.646	0.632
	Progm	**0.865**	0.536	0.447	0.535	0.659	0.654	**0.888**	0.439	0.339	0.576	0.603	0.528	1.000	0.726	0.365	0.665	0.679	0.664
	Compare	0.722	0.570	0.459	0.545	1.000	0.692	0.709	0.463	0.325	0.634	0.652	0.567	0.659	0.765	0.353	0.711	0.738	0.720
	Negemo	0.668	0.605	0.491	0.546	0.652	0.688	0.666	0.460	0.319	**0.827**	1.000	0.582	0.603	0.705	0.343	0.676	0.672	0.650
	Social	0.742	0.581	0.450	0.590	0.711	**0.758**	0.746	0.500	0.421	0.652	0.676	0.603	0.665	**0.778**	0.456	1.000	0.733	0.705
	Male	0.346	0.296	0.257	0.290	0.325	0.346	0.352	0.253	1.000	0.308	0.319	0.300	0.339	0.339	0.790	0.421	0.338	0.330
	Insight	**0.871**	0.578	0.473	0.595	0.709	0.720	1.000	0.488	0.352	0.634	0.666	0.574	0.888	0.785	0.383	0.746	0.733	0.704
	Bio	0.587	1.000	0.774	0.457	0.570	0.589	0.578	0.390	0.296	0.537	0.605	0.501	0.536	0.603	0.320	0.581	0.584	0.565
	Body	0.467	**0.774**	1.000	0.371	0.459	0.457	0.473	0.307	0.257	0.436	0.491	0.402	0.447	0.474	0.278	0.450	0.461	0.459
	Drives	0.732	0.589	0.457	0.573	0.692	1.000	0.720	0.460	0.346	0.664	0.688	0.790	0.654	0.781	0.364	0.758	0.751	0.694
	Power	0.580	0.501	0.402	0.476	0.567	**0.790**	0.574	0.373	0.300	0.553	0.582	1.000	0.528	0.641	0.317	0.603	0.652	0.567
	Relativ	**0.800**	0.603	0.474	0.608	**0.765**	**0.781**	**0.785**	0.494	0.339	0.671	0.705	0.641	0.726	1.000	0.368	0.778	0.898	0.825
	Space	0.745	0.584	0.461	0.571	0.738	0.751	0.733	0.475	0.338	0.646	0.672	0.652	0.679	**0.898**	0.364	0.733	1.000	0.735
	Time	0.710	0.565	0.459	0.555	0.720	0.694	0.704	0.444	0.330	0.632	0.650	0.567	0.664	**0.825**	0.359	0.705	0.735	1.000
	Assent	1.000	0.587	0.467	0.582	0.722	0.732	0.871	0.485	0.346	0.635	0.668	0.580	0.865	0.800	0.372	0.742	0.745	0.710

Note. All correlation coefficients are significant at $p < .001$. 'S1P1' refers to High SFM and High POM. 'S1P0' refers to High SFM and Low POM. The 15 strongest correlations between categories are shown in boldface, corresponding to thicker edges and shorter inter-node distances in Figures F1.

Figure F1. Sub-network structures of MIL: Low SFM & Low POM, Low SFM & High POM, High SFM & Low POM, and High SFM & High POM.

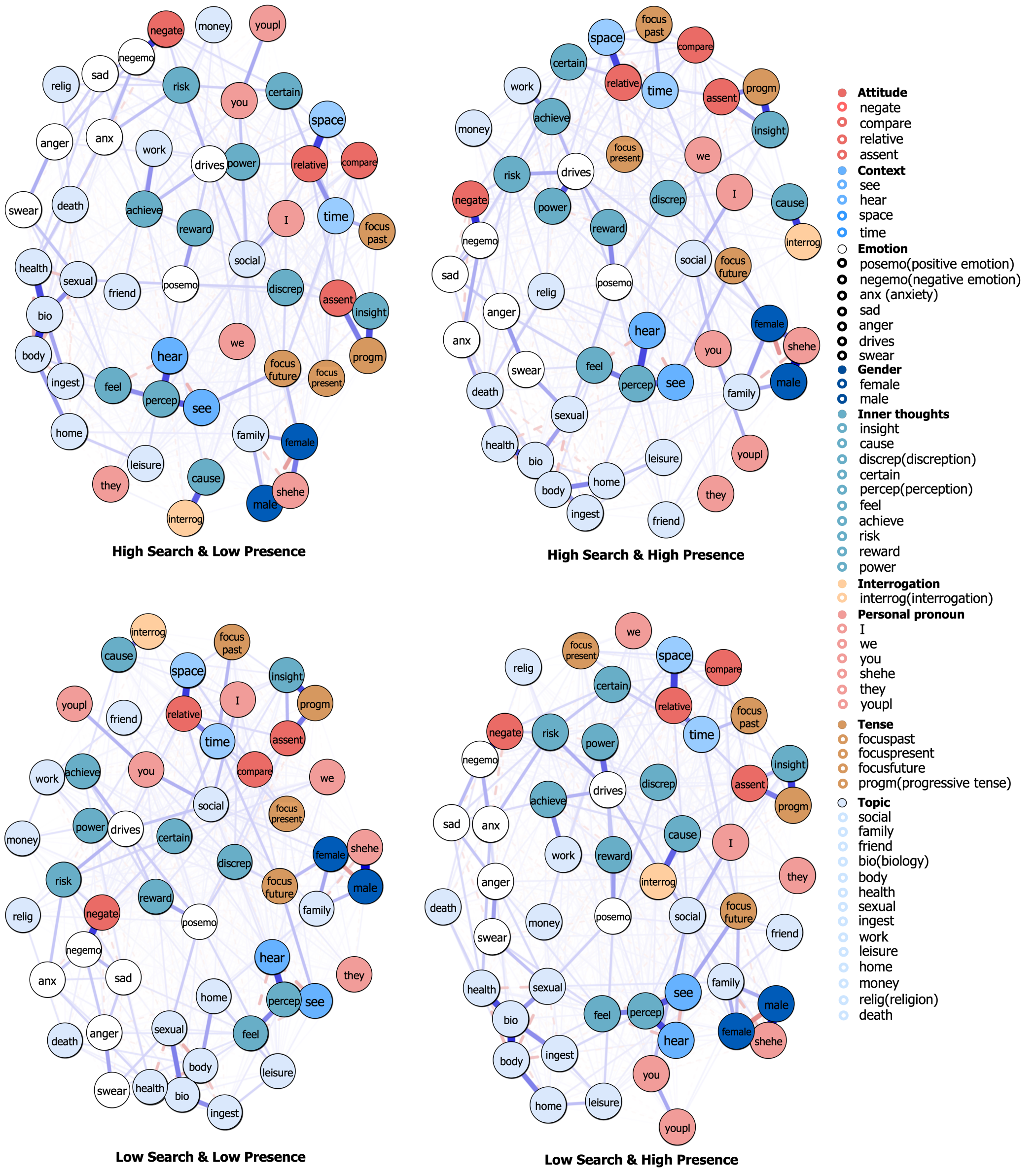

Note. Each node corresponds to one of the 52 LIWC categories, aggregated into nine groups, each shown in a different color. Thicker edges indicate greater edge strength, showing a stronger connection between two MIL-associated factors within the current subnetwork.

Figure F2. Sub-network structures of MIL: Low SFM, Low POM, High SFM, and High POM.

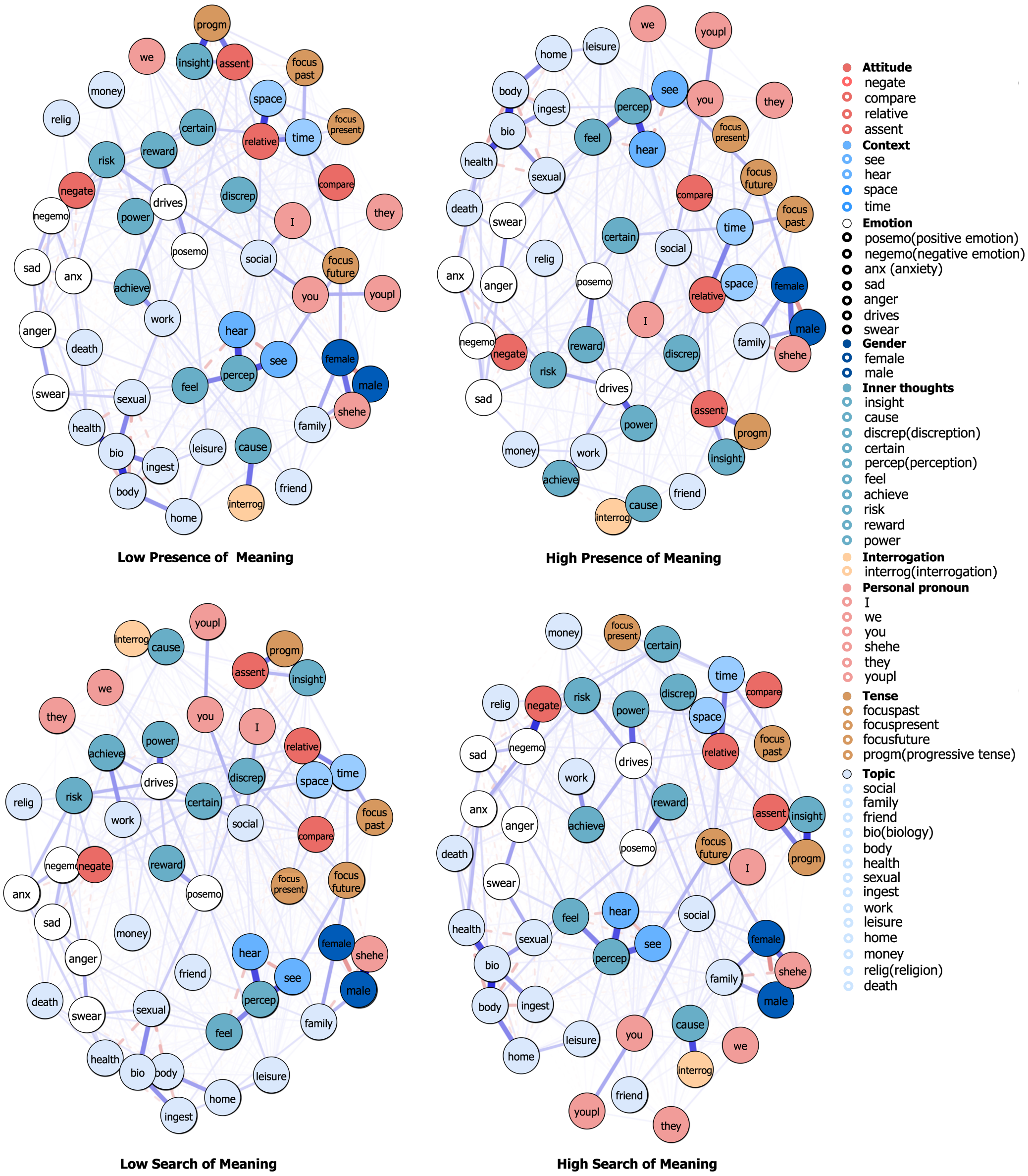

Note. Each node corresponds to one of the 52 LIWC categories, aggregated into nine groups, each shown in a different color. Thicker edges indicate greater edge strength, showing a stronger connection between two MIL-associated factors within the current subnetwork.

Figure F3. Community distributions across the four subnetworks of MIL.

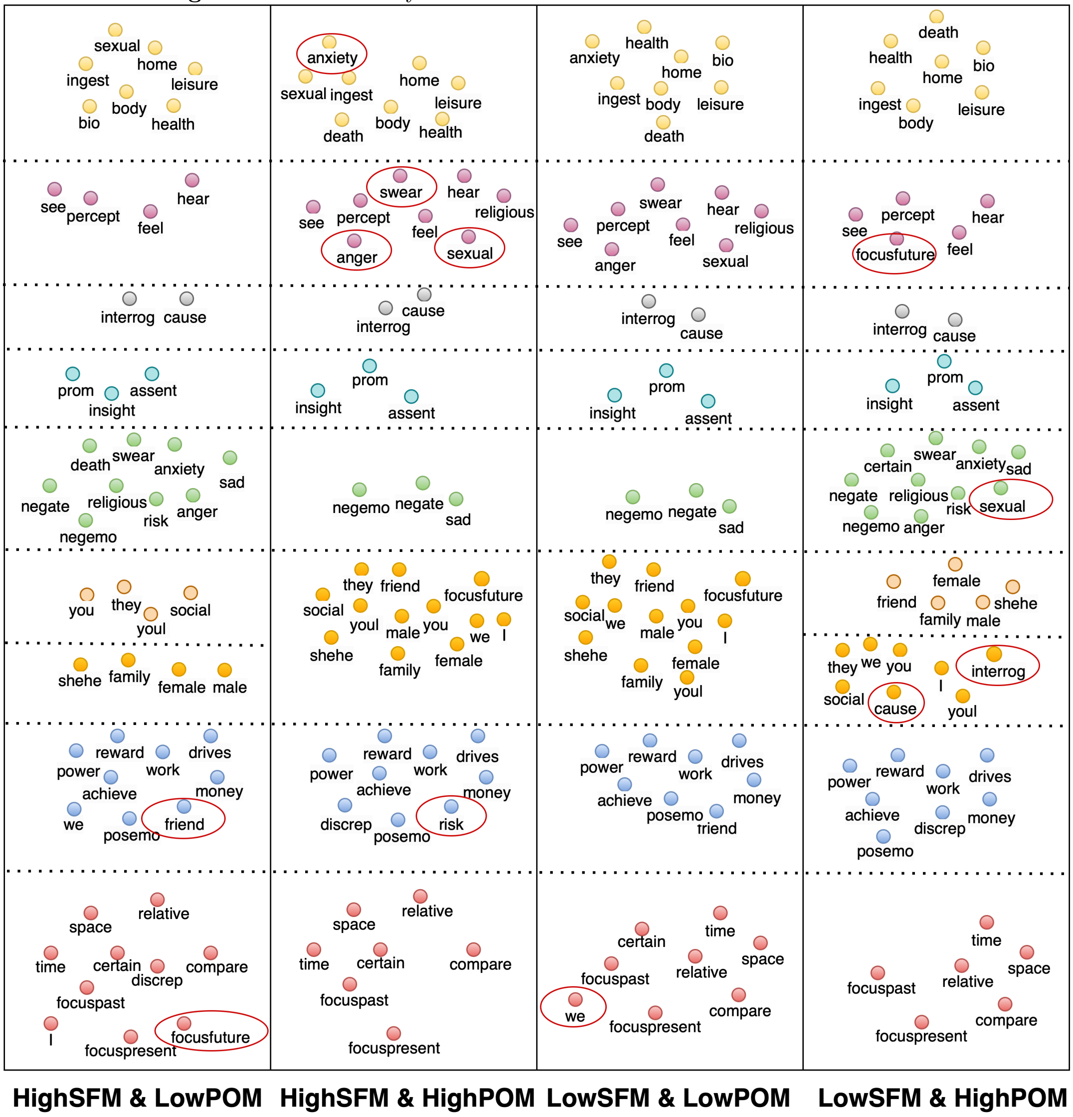

Note. Each community denotes a group of MIL-associated factors that are more densely connected to one another than to factors outside the group. For example, "space" and "time" appear in different clusters: in SFM High & POM Low, "space" and "time" cluster with "focus past", "focus present", and "focus future", whereas in SFM Low & POM Low they cluster only with "focus past" and "focus present".
